# PRMT3 drives glioblastoma progression by enhancing HIF1A and glycolytic metabolism

**DOI:** 10.1038/s41419-022-05389-1

**Published:** 2022-11-09

**Authors:** Yunfei Liao, Zaili Luo, Yifeng Lin, Huiyao Chen, Tong Chen, Lingli Xu, Sean Orgurek, Kalen Berry, Monika Dzieciatkowska, Julie A. Reisz, Angelo D’Alessandro, Wenhao Zhou, Q. Richard Lu

**Affiliations:** 1 Key Laboratory of Birth Defects, Children’s Hospital, Institutes of Biomedical Sciences, Fudan University, Shanghai, China; 2 Brain Tumor Center, Division of Experimental Hematology and Cancer Biology, Cincinnati Children’s Hospital Medical Center, Cincinnati, OH 45229 USA; 3 University of Colorado Anschutz Medical Campus, Aurora, CO 80045 USA

**Keywords:** CNS cancer, Cancer stem cells

## Abstract

Glioblastoma (GBM) is the most common and aggressive primary brain tumor, but the mechanisms underlying tumor growth and progression remain unclear. The protein arginine methyltransferases (PRMTs) regulate a variety of biological processes, however, their roles in GBM growth and progression are not fully understood. In this study, our functional analysis of gene expression networks revealed that among the PRMT family expression of PRMT3 was most significantly enriched in both GBM and low-grade gliomas. Higher PRMT3 expression predicted poorer overall survival rate in patients with gliomas. Knockdown of PRMT3 markedly reduced the proliferation and migration of GBM cell lines and patient-derived glioblastoma stem cells (GSC) in cell culture, while its over-expression increased the proliferative capacity of GSC cells by promoting cell cycle progression. Consistently, stable PRMT3 knockdown strongly inhibited tumor growth in xenograft mouse models, along with a significant decrease in cell proliferation as well as an increase in apoptosis. We further found that PRMT3 reprogrammed metabolic pathways to promote GSC growth via increasing glycolysis and its critical transcriptional regulator HIF1α. In addition, pharmacological inhibition of PRMT3 with a PRMT3-specific inhibitor SGC707 impaired the growth of GBM cells. Thus, our study demonstrates that PRMT3 promotes GBM progression by enhancing HIF1A-mediated glycolysis and metabolic rewiring, presenting a point of metabolic vulnerability for therapeutic targeting in malignant gliomas.

## Introduction

Glioblastoma (GBM) is the most prevalent and aggressive primary brain malignancy in adults, exhibiting a very poor prognosis with a median survival of only around 15 months despite aggressive treatments [1, 2]. Intratumoral cellular heterogeneity and plasticity as well as the infiltrative and migratory nature of tumor cells have been shown to contribute to tumor recurrence and the poor prognosis for glioblastoma patients [3]. GBM stem-like cells (GSCs) have been proposed to be responsible for therapy resistance and tumor recurrence [3, 4]. At present the genetic and epigenetic pathways that regulate the growth and plasticity of GSCs remain poorly understood.

Epigenetic post-translational modifications such as protein methylation and acetylation are critical for the activities of key signaling regulators of brain tumorigenesis [5, 6]. Protein arginine methylation, a common post-translational modification that regulates a variety of cellular functions, is catalyzed by a family of protein arginine methyltransferases (PRMTs), which transfer a methyl group from S-adenosyl-methionine (SAM) to the arginine residues of protein substrates [7]. Nine PRMTs (PRMT1-9) have been identified in mammals [7–9] and can be divided into three types based on the arginine methylation they catalyzed [10]. Type I PRMTs (PRMT1, PRMT2, PRMT3, CARM1/PRMT4, PRMT6, PRMT8) catalyze asymmetric dimethylation of arginine residues, while type II PRMTs (PRMT5 and PRMT9) predominantly catalyze symmetric dimethylation, and type III PRMTs (PRMT7) mediate monomethylation [11]. PRMT family members have been shown to regulate various biological processes, including tumorigenesis [12–18]. Among Type I PRMTs, PRMT2 promotes tumor growth by asymmetrically methylating histone H3R8 (H3R8me2a), which is enriched in promoters and enhancers correlated with known active histone marks, to maintain or activate expression of oncogenic genes in GBM cells [12]. PRMT6 has been shown methylate RCC1 (regulator of chromatin condensation 1) at arginine 214 to facilitate RCC1 association with chromatin and RAN-GTPase activation, which enhances mitotic activity in GSCs and facilitates nucleocytoplasmic transport during interphase, thereby promoting GBM cell proliferation and therapy resistance [13]. In addition, knockdown of PRMT1 can lead to the G1-S phase arrest of the cell cycle, proliferation inhibition and apoptosis induction in glioma cells in vitro and in xenografts, suggesting a potential oncogenic role for PRMT1 in gliomas [19]. Similarly, inhibition of Type II PRMT5 disrupts the alternative splicing of detained introns, leading to cell cycle defects and cell death in GBM cells [15, 16]. These studies suggest a critical role of protein arginine methylation in GBM progression and treatment resistance. Among the PRMT family, PRMT3, a type I PRMT family member, contains a unique C2H2 zinc finger domain [20–22], and is important for the maturation of the 80S ribosome by catalyzing the methylation of the 40S ribosomal protein S2 (RPS2) [21, 23–26]. Although PRTM3 is widely distributed in different cell types, its biological functions in GBM tumorigenesis remain unknown.

In this study, we explored the expression levels and prognostic values of PRMT genes in gliomas using Gene Expression Profiling Interactive Analysis (GEPIA) [27] and found that PRMT3 is the most significantly enriched member of the PRMT family in high- and low-grade gliomas. PRMT3 expression levels were negatively correlated with the survival of GBM patients. Loss- and gain-of-function analyses indicated that PRMT3 is critical for GBM growth by regulating cell cycle progression and cell survival. PRMT3 deficiency inhibited tumor formation in vivo and prolonged mouse survival. Furthermore, we found that PRMT3 promotes GBM growth at least in part by maintaining HIF1A stability and thus promoting the expression of its downstream target glycolytic enzymes. Pharmacological inhibition of PRMT3 with a PRMT3 inhibitor SGC707 abolished GBM glycolysis and tumor growth. Thus, our data demonstrate that PRMT3 exhibits a critical oncogenic role for GBM growth, and might serve as a promising potential therapeutic target for GBM.

## Results

### PRMT3 is highly expressed in GBM patients and negatively correlated with prognosis

**Fig. 1:**
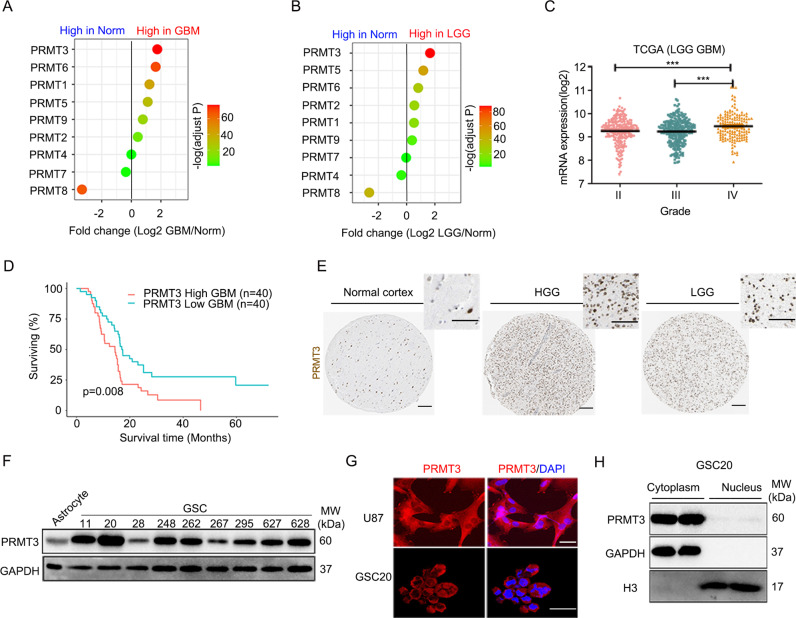
PRMT3 is overexpressed in GBM and negatively correlated with prognosis. **A**, **B** The differential expression of PRMT genes in the TCGA GBM (**A**) and LGG (**B**) datasets. **C** PRMT3 expression levels in grade IV GBMs are the highest among TCGA gliomas. **D** Kaplan-Meier survival curve for PRMT3 in GBMs. Figure was created using GlioVis data portal (http://gliovis.bioinfo.cnio.es/). **E** Immunostaining of PRMT3 in the normal cerebral cortex, HGG, and LGG tissues from the Human Protein Atlas. Scale bars, 100 μm. **F** Western blot of PRMT3 expression in human astrocytes and GSC11, GSC20, GSC28, GSC248, GSC262, GSC267, GSC295, GSC627 and GSC628 cells. **G**, **H** Immunofluorescence assays (**G**) and western blot (**H**) of GBM cells showing the localization of PRMT3. Scale bars, 25 μm.

To identify PRMTs that are involved in gliomagenesis and progression, we conducted differential expression analysis through GEPIA (http://gepia.cancer‐pku.cn/) to evaluate the expression of PRMTs in GBM and low-grade gliomas (LGG) compared with normal brain tissues. Integrative GEPIA analysis of gliomas from TCGA (The Cancer Genome Atlas) and the human Genotype-Tissue Expression (GTEx) database [28], we found that PRMT3 was the most significantly enriched factor among all the PRMTs (based on fold change and p value) in both GBM and LGG (Fig. 1A, B). The expression level of PRMT3 was also higher in GBM (grade IV) than low-grade gliomas (grades II or III) in the TCGA database (Fig. 1C). Importantly, based on the GEO datasets, we found that high PRMT3 expression levels were associated with poor prognosis in GBM patients (Fig. 1D).

The density of PRMT3-expressing cells in gliomas was higher in LGG and HGG than normal brain tissues based on the Human Protein Atlas data (https://www.proteinatlas.org) (Fig. 1E). Western blotting further indicated that the expression of PRMT3 in multiple GBM cell lines and patient-derived GBM stem cell lines (GSCs) was higher than that in normal human astrocytes as well as in a normal brain glial cell line (HEB) [29] and a microglia cell line (HMO6) [30] (Fig. 1F and Supplementary Fig. 1). To further examine the subcellular localization of PRMT3, we performed immunostaining in a GBM cell line U87 and a patient-derived GSC cell line, GSC20. As shown in Fig. 1G, PRMT3 was mainly localized in the cytoplasm of the glioma cells. We validated the cytoplasmic expression of PRMT3 by a nucleocytoplasmic fractionation assay in GSC20 cells (Fig. 1H). These data indicate that cytoplasm-expressing PRMT3 is highly enriched in GBM and negatively correlated with patient survival.

### PRMT3 is required for the GBM cell growth

**Fig. 2:**
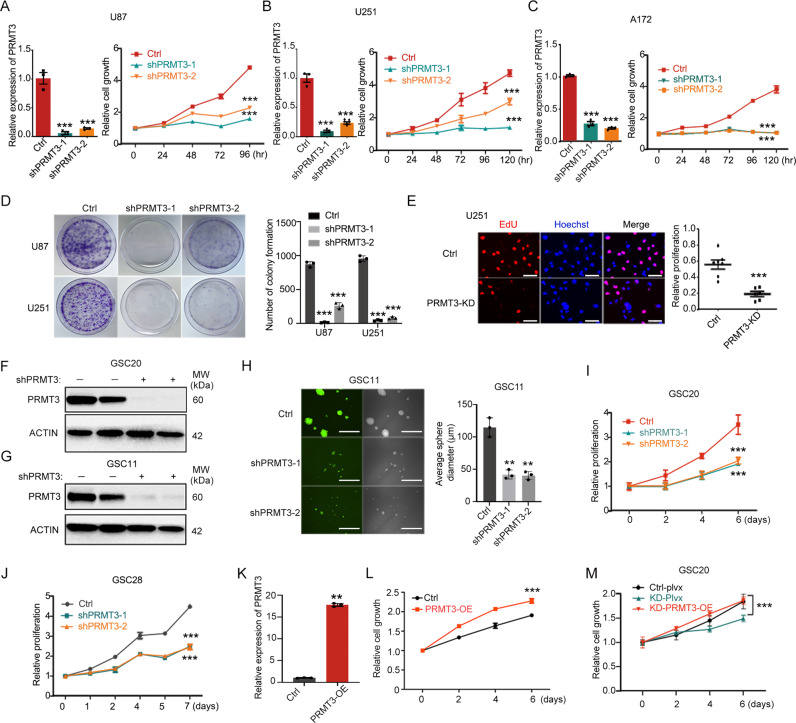
PRMT3 is essential for GBM cell growth in cultures. **A–C** qRT-PCR validation of shPRMT3-mediated *PRMT3* depletion in U87 (**A**), U251(**B**) and A172 (**C**) cells (left) and the growth of U87 (**A**), U251 (**B**) and A172 (C) cells as tested by WST-1 assay (right). Growth curves are calculated at indicated days post-transduction of lenti-control or shPRMT3. Day 0 represents 2 days after PRMT3 knockdown. **D** Images of colonies of wild-type and *PRMT3*-KD U87-MG and U251cells (left). Quantification (right) of relative clones. **E** EdU staining (left) and quantification (right) of wild-type and *PRMT3*-KD U251 cells after 1 hour of EdU treatment. **F**, **G** Western blot validation of shPRMT3-mediated PRMT3 depletion in GSC20 (**F**) and GSC11 (G) cell cultures. ACTIN: loading control. **H** Representative images of spheres (left) and quantification (right) of sphere sizes of GSC11 Ctrl and PRMT3 KD cells. **I**-**J** Cell growth curves of GSC20 (**I**) and GSC28 (**J**) cells treated with control shRNA- or shPRMT3 lenti-virus as determined by WST-1 assay. Day 0 represents the assay start from 2 days after transduction. **K** qRT-PCR validation of lenti-PRMT3 mediated *PRMT3* overexpression in GSC20 cells. **L** Cell growth of GSC20 cells treated with control or lentiviral vector expressing PRMT3 as tested by WST-1 assay. Day 0 represents the assay start from 2 days after transduction. **M** Cell growth of control or PRMT3-KD GSC20 cells treated with control or lentiviral vector expressing PRMT3 as tested by WST-1 assay. Day 0 represents the assay start from 2 days after transduction. Data are presented as means ± SEM; *n* = 3 independent experiments; **p < 0.01; ***p < 0.001; two-tailed unpaired Student t-test (in **E, K** and **L**) or one-way ANOVA with multiple comparison test (in **A**–**D, H**–**J** and **M**).

To determine the role of PRMT3 in the growth of GBM cells, we first knocked down PRMT3 with two short hairpin RNAs (shRNAs) (shPRMT3-1 and shPRMT3-2) in different GBM cell lines (U87, U251, and A172). PRMT3 depletion was confirmed by real-time quantitative PCR (RT-qPCR) and western blot (Fig. 2A-C and Supplementary Fig. 2A-C). Using WST-1 cell viability and proliferation assays, we found that knockdown of PRMT3 resulted in a significant reduction in tumor cell growth and proliferation (Fig. 2A-C). Similarly, PRMT3 knockdown led to a reduction in colony formation in a soft-agar assay (Fig. 2D). Furthermore, EdU pulse labeling assays indicated that the proportion of cells in S phase was reduced in PRMT3-KD cells (Fig. 2E). These observations suggest that PRMT3 is critical for the growth of GBM cells.

To further determine the function of PRMT3 in patient-derived glioblastoma stem cells, we transduced GSC11 and GSC20 with lentiviral vectors carrying shRNAs to knockdown PRMT3. The depletion of PRMT3 was confirmed by western blotting (Fig. 2F-G). We observed that knockdown of PRMT3 in GSC11 cells inhibited tumor-sphere formation as indicated by a reduction in the sphere size (Fig. 2H). In addition, the WST-1 assay indicated that knockdown of PRMT3 significantly inhibited the growth of GSC20 and GSC28 cells (Fig. 2I-J).

In contrast to PRMT3 knockdown, overexpression of PRMT3 markedly promoted the growth of GSC20 and GSC28 cells compared with the control (Fig. 2K-L and Supplementary Fig. 2D-E). To further determine whether PRMT3 overexpression could rescue the proliferation defects in PRMT3-deficient GSCs, we transduced lentivector expressing PRMT3 with mutated shRNA sites in PRMT3-knockdown GSC20 cells. PRMT3 overexpression rescued the growth defect of PRMT3- knockdown cells by WST-1 assays (Fig. 2M), confirming the targeting specificity of PRMT3-knockdown in the GSC cells. Taken together, our loss- and gain-of-function analyses suggest that PRMT3 is both necessary for and sufficient to promote tumor cell growth in GBM.

### PRMT3 deficiency induces cell cycle arrest and cell apoptosis

**Fig. 3:**
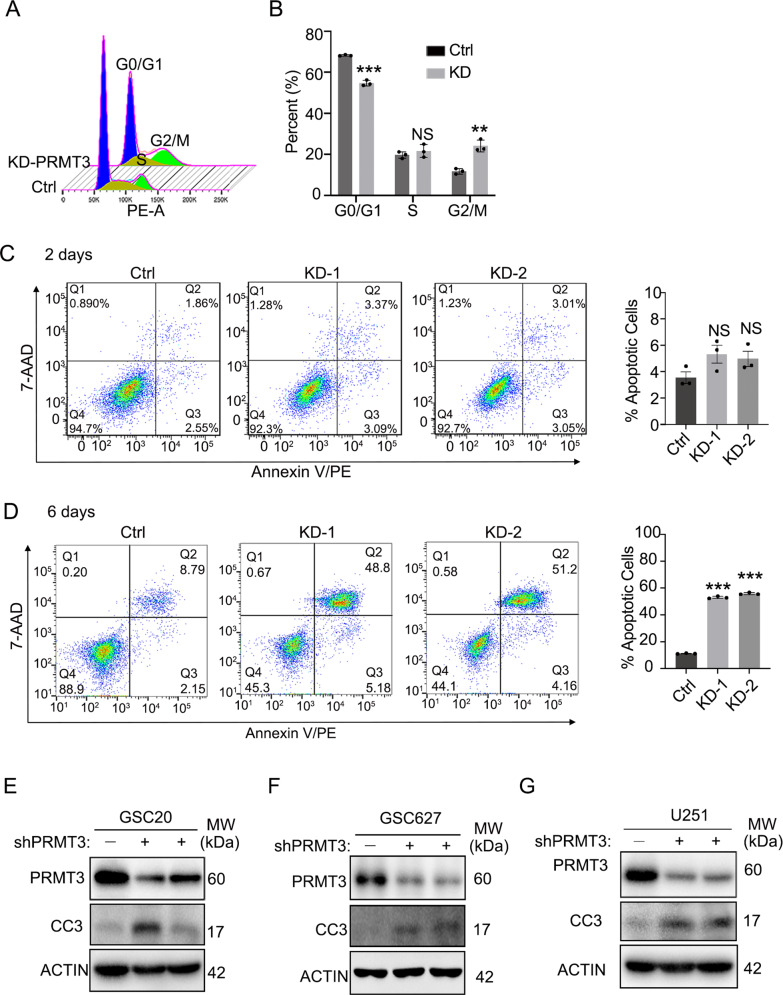
PRMT3 deficiency induces cell cycle arrest and cell death. **A**, **B** Flow cytometry cell cycle analysis (**A**) and quantification (**B**) of shCtrl and shPRMT3-1 (PRMT3 KD) GSC20 cell cultures. Cells were transduced with control or shPRMT3-1 lenti-virus after 48 h. **C**, **D** Representative flow cytometry analysis of cell apoptosis (left) and quantification (right). Cells were transduced with control or shPRMT3 lenti-virus after 2 days (**C**) or 6 days (**D**). **E-G** Representative western blots for cleaved-caspase 3 (CC3) and PRMT3 in GSC20 (**E**), GSC627 (**F**) and U251 (**G**) transduced with lenti-shCtrl and lenti-shPRMT3 for 3 days. ACTIN served as a loading control. Data are presented as means ± SEM; *n* = 3 independent experiments; **p < 0.01; ***p < 0.001; two-tailed unpaired Student t-test (in **B**) or one-way ANOVA with multiple comparison test (in **C**, **D**). N.S., not significant.

Previous studies have shown that PRMT3 regulates cell cycle-associated programs in other cellular contexts [31, 32]. We assessed cell cycle progression following PRMT3 knockdown in GSC cells. Cell cycle distribution analysis showed that PRMT3 knockdown induced a G2/M phase arrest in GSC20 cells (Fig. 3A-B). Consistent with the growth inhibition, PRMT3 knockdown also resulted in a decrease in G0/G1 (Fig. 3A-B). In addition, fluorescence-activated cell sorting (FACS) analysis showed that PRMT3 knockdown increased the proportion of apoptotic cells in GSC20 compared with control shRNA-treated cells 6 days after transduction (Fig. 3D), while there was no significant increase in cell death at the early stage 2 days after PRMT3 knockdown (Fig. 3C). Similarly, PRMT3 knockdown also caused cell cycle arrest in the G2/M phase and increased cell apoptosis in U251 GBM cells (Supplementary Fig. 3A-D). Consistently, PRMT3 knockdown led to an upregulation of cell apoptotic markers such as cleaved-caspase 3 and cleaved-PARP1 in GSC20 and GSC627 cells (Fig. 3E,F and Supplementary Fig. 3E,F). Similarly, we detected an upregulation of cleaved-caspase 3 and p21, which promotes cell cycle arrest [33], in PRMT3-knockdown U251 GBM cells (Fig. 3G and Supplementary Fig. 3G). These studies indicate that PRMT3 loss-of-function induces mitotic disruption and apoptosis in GBM cells.

### PRMT3 regulates GBM cell migration capacity

**Fig. 4:**
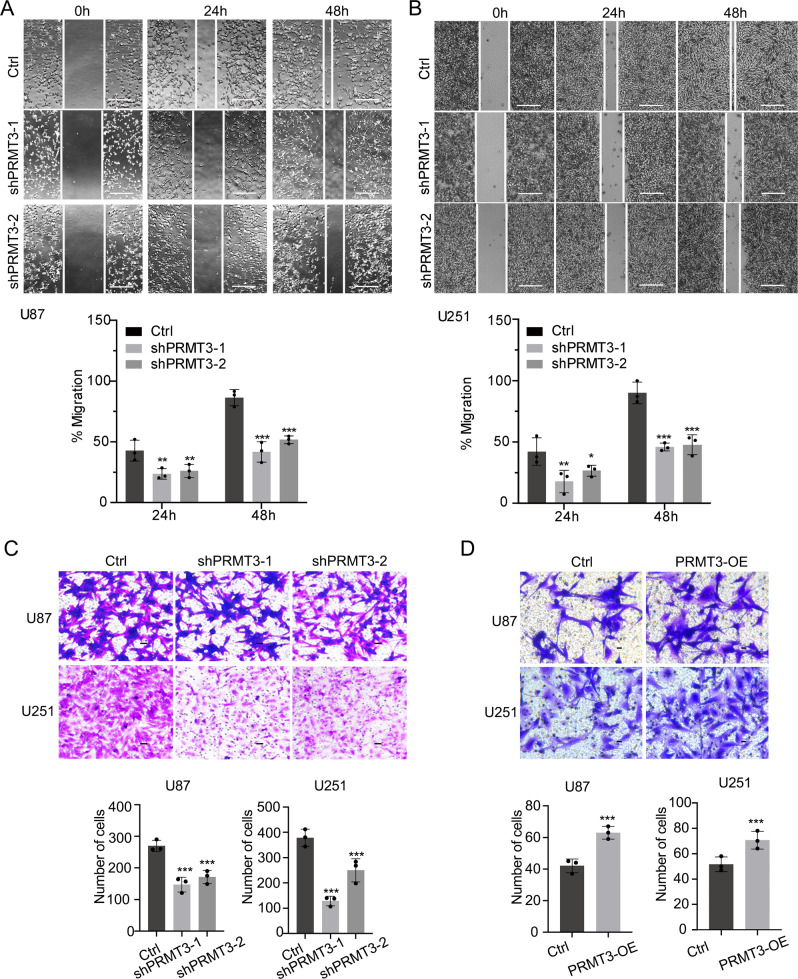
PRMT3 is required for GBM cell migration. **A**, **B** Scratch assays (upper) and quantification (bottom) of Ctrl and PRMT3-KD U87 (**A**) and U251 (**B**) cell lines. **C** Representative images of migrating cells of U87 or U251 treated with Ctrl and PRMT3 shRNA with 48 hr followed by the Boyden chamber invasion assay for 72 hours. Area per field, 1 mm^2^. Scale bar, 100 μm (upper). Quantification of invasive cells of U87 or U251Ctrl and PRMT3 KD cells (bottom). **D** Upper, representative images of migrating cells of U87 or U251 transduced with Ctrl and PRMT3-OE following 72 hours in the Boyden chamber invasion assay; Bottom, Quantification of invasive cells. Area per field, 1 mm^2^. Scale bar, 50 μm. Data are presented as means ± SEM; *n* = 3 independent experiments; *p < 0.05; **p < 0.01; ***p < 0.001; two-tailed unpaired Student t-test (in **D**) or one-way ANOVA with multiple comparison test (in **A**–**C**).

PRMT3 has been shown to promote the migration and invasion of tumor cells [32]. To determine whether PRMT3 could regulate the migration of GBM cells, we used an in vitro scratch assay under a serum-free condition to exclude cell proliferation effects [34, 35]. After PRMT3 knockdown for 24 and 48 h, cell migration was significantly inhibited in U87 cells, with proportions of cells in the wound area of ~45% and ~85% in the control cells compared to ~25% and ~50% in PRMT3-depleted U87 cells, respectively (Fig. 4A). A similar defect in cell migration was also detected in U251 cells with PRMT3 knockdown (Fig. 4B).

The involvement of PRMT3 in GBM cell migration was further confirmed in a transwell plate assay [36]. Depletion of PRMT3 compromised the migration of U87 and U251 GBM cells to the lower surface of the membrane in transwells (Fig. 4C). Conversely, overexpression of PRMT3 increased the migration of U87 and U251 cells (Fig. 4D). Together, these data suggest that PRMT3 regulates the migration capacity of GBM cells.

### PRMT3 is required for GBM tumor growth and progression in vivo

**Fig. 5:**
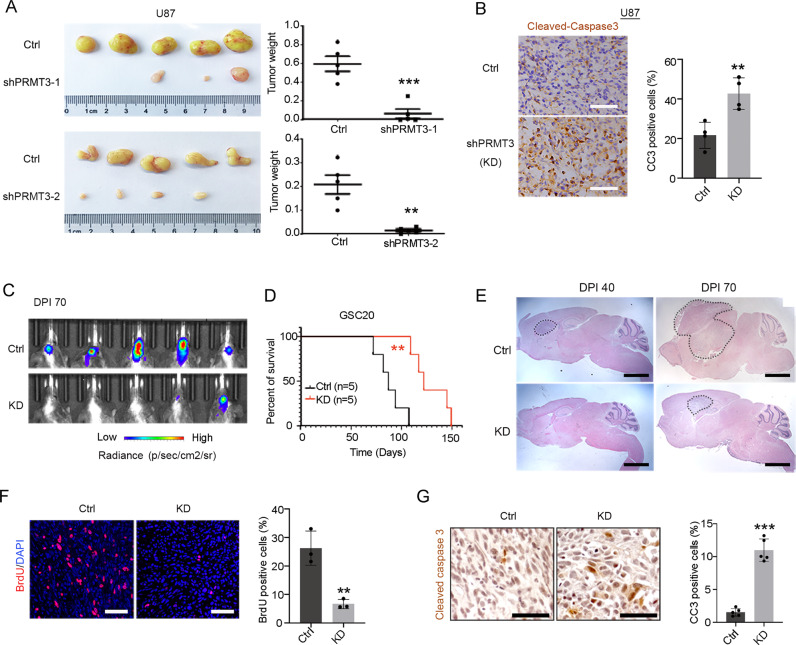
PRMT3 is required for the GBM tumor formation and progression in vivo. **A** Representative images (left) and weights (right) of tumors formed in the flanks of nude mice injected with GBM cells that were transfected with control or lenti-shPRMT3. Data are presented as means ± SEM; *n* = 5 animals/group. **p < 0.01; ***p < 0.001; two-tailed unpaired Student t-test. **B** Tumors stained for cleaved-caspase3 from mice injected with U87 cells that were transfected with control or lenti-shPRMT3 (left) and quantifications (right) of CC3 positive cells. Data are presented as means ± SEM; *n* = 4 independent experiments; **p < 0.01; ***p < 0.001; two-tailed unpaired Student t-test. **C** Representative bioluminescent images of mice transplanted with Ctrl or PRMT3-KD GSC20 cells. Bar graph: average photon flux at each time point. **D** Kaplan-Meier survival curve of mice. Log-rank test. **E** Representative images of tumors stained with H&E. **F** Representative images of tumors stained with BrdU (left) and quantifications (right) of BrdU positive cells. Data are presented as means ± SEM; *n* = 3 independent experiments; **p < 0.01; ***p < 0.001; two-tailed unpaired Student t-test. **G** Tumors stained for cleaved-caspase3 from mouse xenografts with GSC20 cells that were transduced with control or lenti-shPRMT3 (left) and quantifications (right) of CC3 positive cells. Data are presented as means ± SEM; *n* = 5 animals/group. **p < 0.01; ***p < 0.001; two-tailed unpaired Student t-test.

To further define the role of PRMT3 in GBM growth in vivo, we first subcutaneously transplanted U87 cells with stable PRMT3 knockdown into nude mice. The tumor volumes and weights were significantly reduced in the PRMT3 knockdown groups when compared with controls (Fig. 5A). Moreover, immunostaining of cleaved-caspase 3 showed a significant increase in apoptotic cells within PRMT3-knockdown tumors, suggesting that PRMT3 knockdown induces tumor cell apoptosis in the subcutaneous model (Fig. 5B).

To further examine the effect of PRMT3 loss in orthotopic PDX models, we performed intracranial implantation of GSC20 cells treated with control shRNA- or shPRMT3, which carried a luciferase reporter, into the cortex of immunocompromised NSG mice. In contrast to the control group, the mice implanted with PRMT3-depleted GSC 20 cells exhibited a significant decrease in tumor cell growth at Day 70 post-transplantation and a significant extension of lifespan (Fig. 5C, D). Histology analysis indicated that PRMT3-knockdown tumors exhibited a reduced tumor mass (Fig. 5E). The BrdU incorporation assay further indicated a reduction of BrdU-labeled cells in PRMT3-KD tumors compared to control tumors (Fig. 5F). Furthermore, immunostaining staining showed a significant increase in cleaved-caspase 3 expression in orthotopic tumor xenografts with PRMT3-depleted GSC 20 cells (Fig. 5G). Together, these observations suggest that PRMT3 is required for GBM cell growth in vivo.

### PRMT3 deficiency decreases GBM progression by inhibiting aerobic glycolysis

**Fig. 6:**
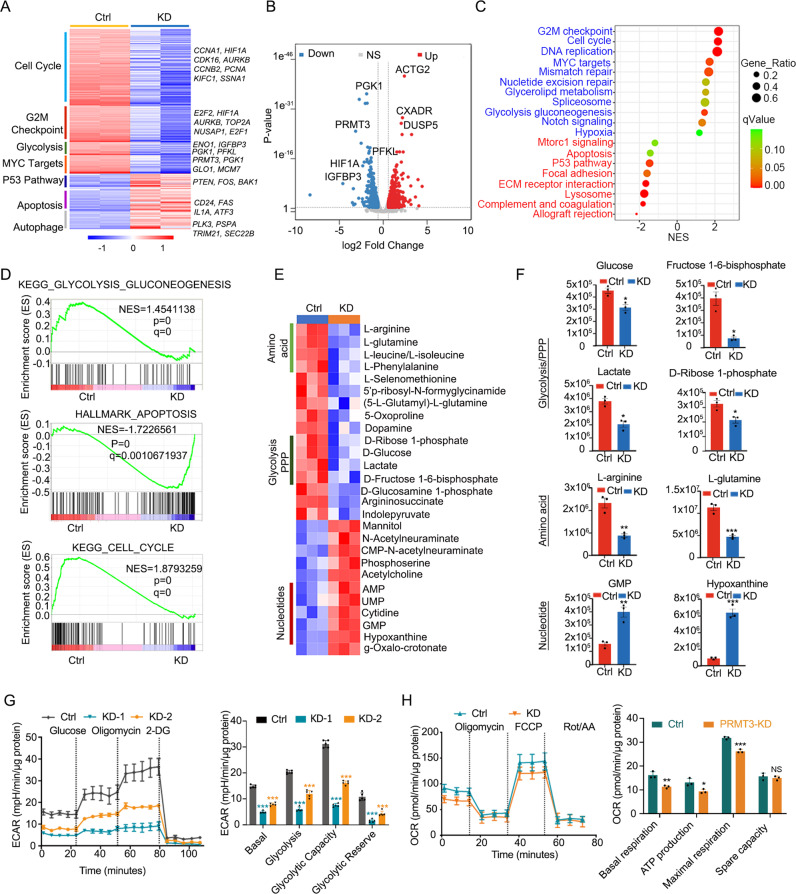
PRMT3 deficiency decreases GBM progression by inhibiting aerobic glycolysis. **A** Heatmap of the genes differentially expressed in control and PRMT3-KD U251cells after 72 hr. **B** Volcano plot of transcriptome profiles of wild-type and PRMT3-KD U251cells. Red and blue dots represent genes significantly upregulated or downregulated, respectively in PRMT3-KD cells compared to wild-type cells (fold change >1.5, *p* < 0.05). **C** KEGG analysis of the upregulated and downregulated genes in PRMT3-KD compared with wild-type U251cells. **D** GSEA enrichment plots of the glycolysis pathway, apoptosis and cell cycle pathway showing the comparison of gene expression profiles in wild-type U251cells and PRMT3-KD cells. NES, normalized enrichment score; *p* value, represents the statistical significance of the enrichment score. **E** Heatmap of the metabolites changed in PRMT3-KD GSC20 cells. **F** Relative levels of the metabolites in glycolysis/PPP, amino acid and nucleotide metabolism in Ctrl (red) and PRMT3-KD (blue) GSC20 cells. Data are presented as means ± SEM; *n* = 3 independent experiments; *p < 0.05; **p < 0.01; ***p < 0.001; two-tailed unpaired Student t-test. **G** ECAR was measured with a Seahorse XF96 Flux analyzer (left) and quantification (right) in Ctrl and PRMT3-KD GSC20 cells. *p < 0.05, **p < 0.01, ***p < 0.001. one-way ANOVA with multiple comparison test. **H**. OCR was measured with a Seahorse XF96 Flux analyzer (left) and quantification (right) in Ctrl and PRMT3-KD GSC20 cells. *p < 0.05, **p < 0.01, ***p < 0.001. two-tailed unpaired Student t-test. N.S., not significant.

To further investigate the potential mechanisms underlying PRMT3 regulation of GBM growth, we performed RNA-seq transcriptomic profiling of U251 GBM cells treated with lenti-shCtrl and shPRMT3. Knockdown of PRMT3 in the GBM cells significantly altered the expression of approximately 1500 genes (>1.5-fold change, *P* value < 0.05) (Fig. 6A, B). There was no significant change in the expression of other PRMT family members after PRMT3 knockdown (Supplementary Fig. 5). The genes associated with the cell cycle (*CCNA1, HIF1A, CCNB2, AURKB, PCNA, SSNA1*), MYC targets (*PRMT3, PGK1, GLO1, MCM7*), and glycolytic pathways (*ENO1, GAPDH, PKM2, IGFBP3, PGK1, PFKL*) were downregulated in PRMT3-knockdown cells, whereas the genes associated with cell apoptosis (*CD24, FAS, IL1A, ATF3*) and p53 signaling (*PTEN, FOS, BAK1*) pathways were upregulated in PRMT3-depleted cells (Fig. 6A). Consistently, gene set enrichment analysis (GSEA) revealed that the cell cycle and glycolysis genes were significantly downregulated, while the apoptosis-associated genes were upregulated after PRMT3 knockdown (Fig. 6C, D).

To further assess the function of PRMT3 in the regulation of metabolic programs in GBM cells, we performed metabolomics analyses by ultra-high pressure liquid chromatography coupled to high-resolution mass spectrometry (UHPLC-HRMS) [37] using GSC20 cells treated with control shRNA- or shPRMT3 (Supplementary Table 1). Metabolomics analyses revealed that PRMT3 knockdown downregulated multiple metabolic pathways, including glycolysis, the pentose phosphate pathway (PPP), and amino acid biosynthesis pathways, while upregulating nucleotide metabolic pathways (Fig. 6E, F).

To further assess the role of PRMT3 in aerobic glycolysis, we knocked down PRMT3 in GSC20 cells and performed a Seahorse real-time cell metabolic analysis to interrogate key cellular functions such as mitochondrial respiration and glycolysis. Silencing PRMT3 significantly reduced glycolysis, glycolysis capacity, and glycolysis reserve in GSC20 cells (Fig. 6G). Consistently, knockdown of PRMT3 also decreased ATP production and the maximum respiration rate, as measured by the oxygen consumption rate (OCR) assay (Fig. 6H). These data indicate that PRMT3 depletion impairs glycolytic metabolism and mitochondrial respiration in GBM cells.

### PRMT3 interacts with HIF1A and regulates its expression

**Fig. 7:**
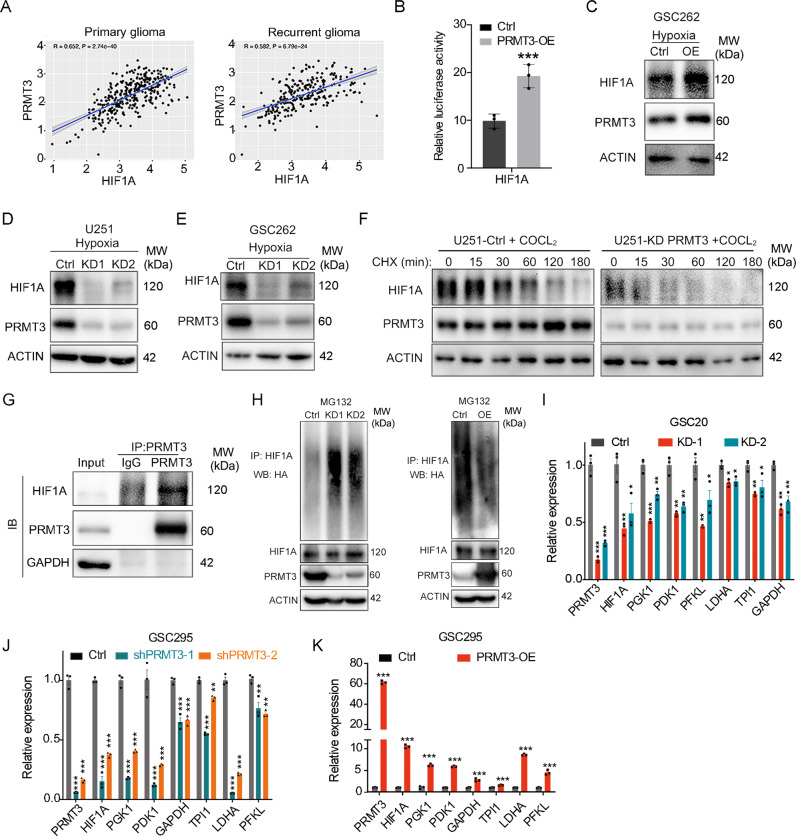
PRMT3 binds and regulates the expression and function of HIF1A. **A** Plot showing the correlation between the mRNA levels of PRMT3 and HIF1A in the CCGA database. r, correlation score; *p* value, statistical significance of the correlation score. **B** Dual luciferase assay for HIF1A reporter activity. Data are presented as means ± SEM; *n* = 3 independent experiments; ***p < 0.001; two-tailed unpaired Student t-test. **C** Representative western blots for HIF1A and PRMT3 in U251Ctrl cells and PRMT3-OE cells under hypoxia. ACTIN served as a loading control. **D**, **E** Representative western blots for HIF1A and PRMT3 in U251 (**D**) and GSC262 (**E**) Ctrl cells and PRMT3-KD cells under hypoxia. ACTIN served as a loading control. **F** The half-life of endogenous HIF-1α detected by western blot after treatment with cycloheximide (CHX) under hypoxia. **G** CO-IP assay for the interaction of endogenous PRMT3 and HIF1A in the GSC262 cell line under hypoxia. **H** Representative western blots for poly-ubiquitin levels of HIF1A in 293 T PRMT3-KD (left) and PRMT3 overexpression (OE) (right) cells. ACTIN served as a loading control. **I**, **J** qRT-PCR analysis of the indicated genes in Ctrl and PRMT3-KD GSC20 (**I**) and GSC295 (**J**) cell lines. Data are presented as means ± SEM; *n* = 3 independent experiments; *p < 0.05, **p < 0.01; ***p < 0.001; one-way ANOVA with multiple comparisons test. **K** qRT-PCR analysis of the indicated genes in Ctrl and PRMT3-OE GSC295 cells. Data are presented as means ± SEM; *n* = 3 independent experiments; ***p < 0.001; two-tailed unpaired Student t-test.

HIF1A is a key regulator of the glycolysis metabolism [4]. Our transcriptomic profiling showed that PRMT3 depletion led to downregulation of *HIF1A* along with glycolytic pathway genes in GBM cells (Fig. 6A, B). The analysis of the CGGA glioma genome database [38] revealed a positive correlation between expression of *PRMT3* and *HIF1A* in both primary and recurrent gliomas (Fig. 7A). We found that PRMT3 overexpression upregulated the activity of *HIF1A*-promoter driven luciferase in 293T cells and HIF1A expression in GSC262 cells assayed by western blotting under hypoxic conditions (Fig. 7B, C). In contrast, PRMT3 knockdown significantly decreased HIF1A expression under hypoxia conditions (1% O2 or CoCl_2_-induced hypoxia [39]), which elevates HIF1A expression [40] in U251 and GSC262 GBM cells assayed by western blotting (Fig. 7D, E). Given that expression of HIF1A is regulated by protein stability [41, 42], we then measured the half-life of HIF1A after cycloheximide (CHX)-treatment to block protein synthesis. As shown in Fig. 7F, PRMT3 knockdown decreased the half-life of endogenous HIF1A. Furthermore, co-immunoprecipitation analysis showed a physical interaction between PRMT3 and HIF1A in a complex in GSC262 cells under hypoxia conditions (Fig. 7G). HIF1A expression and stability can be modulated by poly-ubiquitin modification [43]. PRMT3 knockdown significantly increased HIF1α poly-ubiquitination, leading to HIF1α de-stabilizattion [43], while overexpression of PRMT3 significantly inhibited HIF1α poly-ubiquitination (Fig. 7H). qRT-PCR analyses showed that PRMT3 deficiency decreased the expression of *HIF1A* and its downstream target glycolytic genes including *PGK1, PDK1, GAPDH, TPI1, LDHA* and *PFKL* (Fig. 7I, J). Conversely, overexpression of PRMT3 promoted the expression of these glycolytic genes (Fig. 7K). These data suggest that PRMTs regulate the glycolysis pathway at least in part by regulating the expression of HIF1A and its protein stability in GBM cells.

### Pharmacological inhibition of PRMT3 inhibits HIF1A expression and glioma growth

**Fig. 8:**
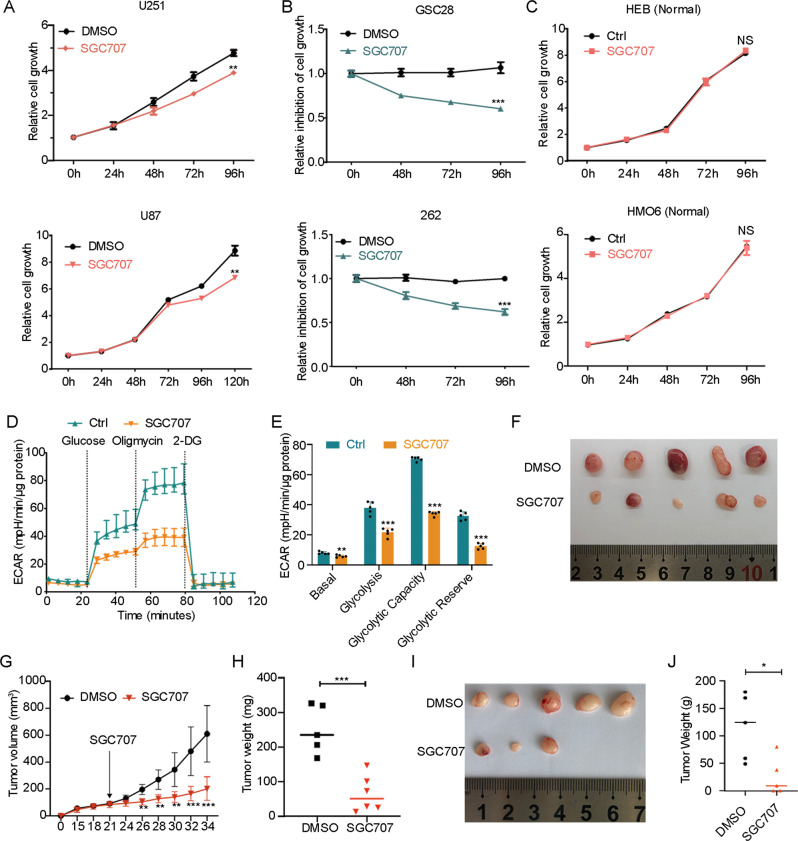
Pharmacological Inhibition of PRMT3 Decreases GBM growth. **A** Growth of U251 and U87 cells treated with SGC707 (10 μM) at the indicated times after treatment initiation. *n* = 3 independent assays. **B** Growth inhibition of GSC28 and GSC262 cells treated with SGC707 (10 μM) at the indicated times after treatment initiation. *n* = 3 independent assays. **C** Growth of HEB and HMO6 normal brain cells treated with SGC707 (10 μM) at the indicated times after treatment initiation. *n* = 3 independent assays. **D**, **E** ECAR of GSC262 cells after treatment with SGC707 for 72 h (**D**) and quantification (**E**). *n* = 5 independent assays. **F** Photomicrographs of tumors formed in the flanks of nude mice injected with GSC262 cells that were treated with DMSO or SGC707. **G** Volumes of tumors formed in the flanks of nude mice injected with GSC262 cells that were treated with DMSO or SGC707. Arrow: treatment starting date. *n* = 5 or 6 animals/group. **H** Weights of tumors formed in the flanks of nude mice injected with GSC262 cells that were treated with DMSO or SGC707. *n* = 5 or 6 animals/group. **I** Photomicrographs of tumors formed in the flanks of nude mice injected with GSC20 cells that were treated with DMSO or SGC707. **J** Weights of tumors formed in the flanks of nude mice injected with GSC20 cells that were treated with DMSO or SGC707. *n* = 5 animals/group. Data are presented as means ± SEM; *p < 0.05, **p < 0.01; ***p < 0.001; two-tailed unpaired Student t-test. N.S., not significant.

We next investigated the effect of pharmacological inhibition of PRMT3 on GBM growth by using the PRMT3-specific inhibitor SGC707. The WST-1 assay showed that treatment of GBM cells (U251, U87) and patient-derived GSC cells (GSC28 and GSC262) with the PRMT3 selective inhibitor SGC707 significantly reduced cell growth (Fig. 8A, B), while it did not affect the cell growth of normal brain glial cell lines, HEB and HMO6, at the same concentration (Fig. 8C). Furthermore, treatment with SGC707 significantly inhibited the extracellular acidification rate (ECAR) as monitored by Seahorse XF96 Extracellular Flux analyzer (Fig. 8D), as well as glycolysis, glycolysis capacity, and glycolysis reserve in GSC262 cells (Fig. 8E), consistent with the PRMT3 knockdown results.

To assess the effect of SGC707 on GBM growth in vivo, we treated the mice bearing flank implanted GSC262 tumor cells with SGC707 (30 mg/kg) [44] daily from day 21, when the tumor became visible, to 34 days post-transplantation. Compared with vehicle-treated mice, tumors in SGC707-treated mice were smaller and grew more slowly (Fig. 8F-H). Consistently, treated with SGC707 also decreased tumor growth in GSC20 xenograft mice model (Fig. 8I, J). Together, these results suggest that pharmacological targeting of PRMT3 decreases HIF1A expression and glycolytic rates in GBM cells, and inhibits GBM growth in a xenograft mouse model.

## Discussion

In the present study, we identify PRMT3 as the most significantly enriched PRMT enzyme family member in high- and low-grade gliomas, and show that the PRMT3 level is correlated with poorer prognosis in GBM patients. Our loss-and gain-of-function studies demonstrated that PRMT3 is required for GBM cell proliferation, survival, and tumor growth in vitro and in vivo. It is not uncommon for a gene to be required for cell growth and survival [45, 46]. Our knockdown and gain-of-function experiments indicate that PRMT3 is critical for both tumor cell growth and survival given that PRMT3 may modify a variety of substrates in different signaling pathways [11, 22, 24, 31, 47]. We further showed that PRMT3 promotes the glycolytic program by elevating HIF1A. Moreover, pharmacological targeting of PRMT3 inhibits GBM cell growth by inhibiting HIF1A expression and glycolysis. Thus, our data demonstrate a key pro-oncogenic role of PRMT3 in GBM progression via enhancing HIF1A and glycolysis.

Glycolysis is a conserved metabolic pathway that converts glucose to pyruvate and generates biomass intermediates such as ATP and NADH to sustain cell growth and survival, including GBM [48–51]. Currently, the functional link between PRMTs and glycolysis in glioma stem cells remains elusive. PRMT3 has been shown to regulate metabolic reprogramming in pancreatic cancers by methylating glycolytic pathway components such as GAPDH and ABCG2 to reprogram cellular metabolism to promote tumor progression [52, 53]. Strikingly, our transcriptomic profiling study indicates that PRMT3 deficiency substantially decreased key glycolysis metabolic pathway genes such as *ENO1, GAPDH, PKM2, IGFBP3, PGK1*, and *PFKL* as well as *HIF1A*, a master regulator of glycolysis by promoting the uptake of glucose and glycolysis [54]. Consistently, our unbiased metabolomic analysis show that PRMT3 knockdown diminishes the expression of glycolytic components in the pentose phosphate pathway. Furthermore, we found that PRMT3 expression is positively correlated with HIF1A in primary and recurrent GBM cases, and that PRMT3 can interact with HIF1A under hypoxic conditions and promote HIF1α expression and its stability. These observations are in keeping with PRMT3 interaction and methylation HIF1α for its activity in colorectal cancer [31], suggesting a conserved role of PRMT3 in regulation of HIF1α and glycolysis in different types of cancers. Together, our results indicate that PRMT3 promotes glycolysis metabolic programs by enhancing HIF1A expression and activity to promote GBM cell growth. *PRMT3* has been identified as a direct target gene of HIF1A, which binds to the promotor/enhancer site marked by activating histone marks H3K4me3 and H3K27ac [55] in the *PRMT3* gene locus in U2OS cells [56] (Supplementary Fig. 4B). Consistently, we found that HIF1A overexpression upregulated *PRMT3* promoter activity in a PRMT3-promoter-driven luciferase assay (Supplementary Fig. 4A). Thus, it is conceivable that HIF1A and PRMT3 form a feedback regulatory loop to amplify PRMT3 expression and further enhance HIF1α transactivation activity, suggesting a regulatory role of PRMT3 in HIF1α signaling.

PRMTs have garnered significant interest as novel targets for anticancer drug development [13, 15, 16]. SGC707, a bioavailable drug, is a selective inhibitor of PRMT3 and potently inhibits its methyltransferase activity in cells [44, 57]. SGC707 has been reported to have an anti-tumor effect in other cancer model [47, 57–59]. Herein, we provide the first evidence that SGC707 has an anticancer effect in GBM. We showed that treatment with SGC707 specifically inhibited GBM and GSC cell growth in part by blocking HIF1A expression and the glycolysis rate in GBM cells, with no effect on normal brain cells. In addition, SGC707 suppressed tumor growth in GBM xenograft models. Given that knockdown and pharmacological inhibition of PRMT3 inhibits GBM cell growth, these results suggest that PRMT3 might serve as a potential target for GBM therapy. In conclusion, we demonstrated that PRMT3, highly enriched in malignant gliomas, is essential for GBM growth in vitro and in vivo at least in part by activating HIF1α and glycolysis signaling. These findings revealed a critical role for PRMT3 in glioma growth and progression and present therapeutic vulnerability to PRMT3-targeted therapy in GBM.

## Materials and methods

### Animal experiments

GBM cells (U251 and U87) transduced with lenti-control (shGFP) and shPRMT3 lentivirus were subcutaneously injected into the flank of nude mice. Four to five week old athymic nude mice were purchased from Shanghai SLRC Laboratory Animal Center and used with approval by the Experimental Animal Care and Use Committees of Fudan University. GSCs treated with shCtrl or shPRMT3 were transplanted into the cortex of the NOD scid gamma (NSG) mice with coordinates 1, 0.5, −2 mm. NSG mice were purchased from the Cincinnati Children’s Hospital Medical Center animal core. For in vivo drug treatment with SGC707 (Selleck, cat# S7832). SGC707 was first dissolved in DMSO and then added to a solution of 30% PEG300 with 65% PBS to make a working solution for mouse intraperitoneal injection. SGC707 was administered at 30 mg/kg via intraperitoneal injection daily starting on Day 21 after tumor cell transplantation. The mice were randomized into individual groups. The animal studies were approved by the IACUC (Institutional Animal Care and Use Committees) of the Cincinnati Children’s Hospital Medical Center, USA.

### Cell culture

The HEK293T cell line and the GBM cell lines U251, U87 and A172 were cultured in DMEM medium with 10% fetal bovine serum (FBS) and 1% penicillin/streptomycin. The GSC cell lines of GSC11, GSC20, GSC262, GSC267, GSC295, GSC28, GSC284 and GSC627 were derived from recurrent GBM specimens as previously described [60]. GSCs were cultured in DMEM/F-12 supplemented with D-Glucose (0.27%), progesterone (20 nM), putrescine dihydrochloride (60 µM), heparin (2 µg/ml), HEPES buffer (5 mM), BSA (0.1%), and 1% Insulin-Transferrin-Selenium (ITS) (Gibco, cat# 41400045). Human astrocyte cells were cultured in DMEM supplemented with N2, 10% fetal bovine serum (FBS) and 1% penicillin/streptomycin. All cells were maintained in a humid incubator with 5% CO_2_ at 37 °C.

### Western blotting

For western blotting, GBM cells were lysed in RIPA lysis buffer containing protease and phosphatase inhibitors (Complete-Mini; Roche-Boehringer). We used antibodies against ACTIN (Invitrogen, cat# MA5-11869), GAPDH (Invitrogen, cat# AM4300), PRMT3 (Abcam, cat# ab191562), HIF1A (Abclonal, cat# A7684), and cleaved-caspas3 (CST, cat# 9661s). HRP conjugated secondary antibodies were used form Jackson ImmunoResearch Laboratories. Uncropped western blots data were presented in Supplementary file 1.

### Cell proliferation and migration assays

For the cell proliferation assay, cells were plated in 96-well plates at a density of 5 × 10^3^ in 100 µl of medium per well in the 96 well plate, or treated with SGC707 (10 μM). 10 μM is the lowest concentration that decreases GBM cell growth and is not toxic to normal cells when examining the effect of a series of SGC707 concentrations on GBM and normal cells. Ten microlites of WST-1 (Takara, cat# MK400) were added into each well and incubated for another 1–3 h at 37 °C. The absorbance was detected by multifunctional microplate reader (BioTek Instruments Inc.) at a wavelength of 450 nm according to the manufacturer’s instructions.

For the wound healing assay to assess cell migration, cells were seeded in 6-well plates and cultured overnight to 95% confluency. Scratch-wounds were made on the plate using a 200 µl sterile pipette tip. Cells were washed twice with PBS to clean up the floating cells. Subsequently, cells were cultured with serum-free DMEM continually for the scratch assay as previously described [35]. Photographs were taken at the indicated times.

The transwell migration assay was evaluated using 24-well transwell migration chambers with 8-μm pore inserts (Corning, Cat # 3422). Briefly, 2 × 10^5^ cells in FBS free media were seeded into the upper chambers. The lower chambers were filled with DMEM media (with 20% FBS). After 72 h of incubation, the lower chamber was fixed in 4% paraformaldehyde and stained with crystal violet. Cells in the lower surface were counted in 6 random fields per insert using ImageJ software (http://rsb.info.nih.gov/ij/).

### Tissue sectioning and staining

Mouse brains were fixed in 4% PFA and were subjected to paraffin embedding and sectioning. Serial 5 µM sections were used for conventional H&E staining, immunohistochemistry staining or immunofluorescence as previously described [60]. For immunohistochemistry staining, sections were incubated with cleaved-caspase3 primary antibody. For BrdU staining, tissue sections were denatured with 0.1 N HCl for 1 h in a 37 °C water bath. After denaturation, the sections were neutralized with 0.1 M Borax at pH 8.5 (Sigma) for 10 min. Sections were washed with 0.3% Triton X-100/1×PBS (wash buffer) 3 times and blocked with 5% normal donkey serum (Sigma-Aldrich) in wash buffer for 1 h at room temperature. All immunofluorescence-labeled images were acquired using a Nikon C2 + confocal microscope.

### ECAR and OCR measurement

The extracellular acidification rate (ECAR) and oxygen consumption rate (OCR) were measured by an extracellular flux (XF96) analyzer (Seahorse Bioscience) using a glycolysis stress test kit (Agilent Technologies, cat#103020-100) or a cell mito stress test kit (Agilent Technologies, cat#103015-100), respectively.

### Real-time quantitative PCR

Total RNA was isolated from the GBM cells using TRIzol (Invitrogen, USA) according to the manufacturer’s protocol. The RNA was reverse transcribed using iScript Reverse Transcription Supermix (BioRad, cat#1708 841). SYBR green PCR mix (BioRad) was used to perform the quantitative RT-PCR. The primer pairs were used as follows: β-Actin forward 5′- AATCGTGCGTGACATTAAGGAG -3′ and reverse 5′- ACTGTGTTGGCGTACAGGTCTT -3′; *PRMT3* forward 5′- GAACCTGCTCGTCATCTA -3′ and reverse 5′- CCATTGCCTGGTAAAGTA -3′; *GAPDH* forward 5′- GGATTTGGTCGTATTGGG -3′ and reverse 5′- GGAAGATGGTGATGGGATT -3′; *LDHA* forward 5′- ACCCAGTTTCCACCATGATT -3′ and reverse 5′- CCCAAAATGCAAGGAACACT -3′; *PFKL* forward 5′- TGGTCGGTGGGTTTGAGG -3′ and reverse 5′- CAGGGACGTTGTTGCTGAT -3′; *PGK1* forward 5′- TGAAGATTACCTTGCCTGTT -3′ and reverse 5′- TCTGCTTAGCCCGAGTGA -3′; *PDK1* forward 5′- TCACCAGGACAGCCAATA -3′ and reverse 5′- CCTCGGTCACTCATCTTCA -3′; *HIF1A* forward 5′- AGTGTACCCTAACTAGCCG -3′ and reverse 5′- CACAAATCAGCACCAAGC -3′; *TPI1* forward 5′- CGTGAAGGACTGGAGCAA -3′ and reverse 5′- CCATAAATGATACGGGTGC -3′.

### Cell cycle and apoptosis analysis

For cell cycle analysis, cells were washed twice with cold PBS, fixed with 75% ethanol and stored at 4 °C overnight. Cells were stained with PI (BD Biosciences, cat#550825) for 15 min at room temperature, and then analyzed using a BD Biosciences System.

For cell apoptosis assays, cells were stained with the PE Annexin V Apoptosis Detection Kit I (BD Biosciences, cat#559763) in accordance the manufacturer’s instructions, and then analyzed on a BD Biosciences flow cytometer.

### RNA-seq analysis

GBM cell RNA was extracted using the RNeasy Mini Kit (Qiagen) and the RNA-seq libraries were prepared using Illumina Preparation Kit and sequenced on a HiSeq 2500 sequencer. RNA-Seq data were analyzed by alignment to hg19 using TopHat with default settings as described previously [61]. Unnormalized gene read counts were generated using Cufflinks (http://cole-trapnell-lab.github.io/cufflinks/). Read count normalization and differential gene expression analysis of count were performed by the DESeq2. We used a threshold of 0.05 for adjusted P-values reported by DEseq2 to identify differentially expressed genes. Heatmap of gene expression was generated using R language (http://www.r-project.org). GO-analysis of gene expression changes was performed using Gene Set Enrichment (GSEA, http://www.broadinstitute.org/gsea/index.jsp). Normalized enrichment score (NES) reflects the degree to which the gene-set is overrepresented at the top or bottom of a ranked list of genes.

#### Metabolomics analyses

Metabolomics were performed as previously described [62]. Briefly, cells were extracted in ice cold lysis solution (5:3:2 MeOH:ACN:H_2_O *v/v/v*) at a 1 million cells/1 ml extraction solution. The samples were vortexed vigorously for 30 minutes at 4 °C. The remaining solids were separated from the extract through centrifugation for 10 minutes at 18,213 x g and 4 °C and discarded. The supernatant was analyzed with ultrahigh-performance liquid chromatography coupled to mass spectrometry (UHPLC-MS — Vanquish and Q-Exactive, Thermo Fisher). The UHPLC was run at a flow rate of 450 µL/min using 5-minute gradients in negative and positive ion polarity modes. Extracts were resolved over a Kinetex C18 column, 150 × 2.1 mm, 1.7 µm particle size (Phenomenex) fitted with a guard column (SecurityGuard^TM^ Ultracartridge–UHPLC C18 for 2.1 mm ID Columns–AJO-8782– Phenomenex). The Q-Exactive mass spectrometer scanned in Full MS mode (2 μscans) from 65 to 950 *m*/*z* at 70,000 resolution, with 4 kV spray voltage, 45 sheath gas, and 15 auxiliary gas. Samples were run in randomized order, with technical mixes interjected throughout the run to validate instrument performance. After untargeted acquisition, the data were converted to.mzXML files using Raw Converter (Scripps Research Institute, La Jolla, California, USA) and a targeted analysis was performed. Metabolite peaks were verified and manually selected using the software Maven (Princeton University, Princeton, New Jersey, USA) against a standard library of over 5,000 compounds, as described [62, 63].

### Quantification and Statistical Analysis

Statistical analysis was performed using two-tailed unpaired Student’s tests or one-way ANOVA with multiple comparisons. Data are shown in dot plots, or histograms as mean ± s.e.m, and *p* < 0.05 was considered to be significant. Significance was set as *p* < 0.05, unless otherwise indicated (*p < 0.05, **p < 0.01, ***p < 0.001). Correlation significance of groups was assessed by Pearson’s correlation coefficient test.

## Supplementary information

Supplementary Figure legend

Supplementary Figure1

Supplementary Figure2

Supplementary Figure3

Supplementary Figure4

Supplementary Figure5

Original Data File

Supplementary Table1

Checklist

## Data Availability

All the high-throughput data have been deposited in the NCBI Gene Expression Omnibus (GEO) under accession number GEO: GSE200902. The uncropped western blotting data has been provided in the Supplementary file 1.
