# Supplementary material for: PRMT3 drives glioblastoma progression by enhancing HIF1A and glycolytic metabolism
Source: Cell Death Dis. 2022 Nov 9;13(11):943. doi: 10.1038/s41419-022-05389-1 (PMC9646854; doi:10.1038/s41419-022-05389-1)
Supplement: Supplementary file 1 — Supplementary Figure legend [file 41419_2022_5389_MOESM1_ESM.docx]

**Supplementary Figure legend:**

**Supplementary Fig. 1: PRMT3 is highly expressed in GBM and GSC cells**

Representative western blot of PRMT3 expression in a normal brain glial cell line (HEB) and a microglia cell line (HMO6), GBM cells (U87, U251 and A172) and GSC cells (GSC262, GSC20). ACTIN served as a loading control.

**Supplementary Fig. 2: PRMT3 is required for GBM cell growth in cultures**

A-C. Western blot validation of shPRMT3-mediated *PRMT3* depletion in U87 (A), U251(B) and A172 (C) cells. ACTIN served as a loading control.

D. Immunofluorescence assays of GSC28 cells showing the localization of PRMT3 in Ctrl and PRMT3 overexpressing cells.

E. The growth curve of GSC28 cells treated with control or lentiviral vector expressing PRMT3 by WST-1 assay.

Data are presented as means ± SEM; n = 3 independent experiments; *** p<0.001; two-tailed unpaired Student t-test.

**Supplementary Fig. 3: PRMT3 downregulation induces cell cycle arrest and cell death**

A-B. Flow cytometry cell cycle analysis and quantification of Ctrl and PRMT3-KD U251 cells. Cells were transduced with lenti-shCtrl or shPRMT3-1 after 48h.

C-D. Flow cytometry analysis of cell apoptosis (C) and quantification (D). Cells were treated with lenti-shCtrl or shPRMT3 for 72h.

E-F. Representative western blots for cleaved PARP1 and PRMT3 in GSC627 (E) and GSC20 (F) treated with lenti-shCtrl and shPRMT3 for 72h. ACTIN served as a loading control.

G. Representative western blot for p21 and PRMT3 in U251cells transduced with lenti-shCtrl and shPRMT3 for 72h. ACTIN served as a loading control.

Data are presented as means ± SEM; n = 3 independent experiments; * p<0.05; ** p<0.01; *** p<0.001; two-tailed unpaired Student t-test (in B) or one-way ANOVA with multiple comparison test (in D).

**Supplementary Fig. 4:** **HIF1A targets the active promotor/enhancer of *PRMT3***

A. Luciferase reporter assay for PRMT3 promoter activity. Data are presented as means ± SEM; n = 3 independent experiments; *** p<0.001; two-tailed unpaired Student t-test.

B. Genome browser view of ChIP-seq signals of HIF1A, H3K27ac and H3K4me3 in the PRMT3 locus in U2OS cells. Highlights indicate regulatory elements (promoter/enhancers).

**Supplementary Fig. 5: PRMT3 downregulation do not affect expression of other PRMTs**

Expression of PRMTs after PRMT3 knockdown in U251 GBM cells for 72 h. Data are presented as means ± SEM; n = 3 independent experiments. ***p<0.001; two-tailed unpaired Student t-test.

**Supplementary Table1:** Metabolomic analysis of control and PRMT3-deficient GSC
