# Supplementary figures and images for: PRMT3 drives glioblastoma progression by enhancing HIF1A and glycolytic metabolism
Source: Cell Death Dis. 2022 Nov 9;13(11):943. doi: 10.1038/s41419-022-05389-1 (PMC9646854; doi:10.1038/s41419-022-05389-1)

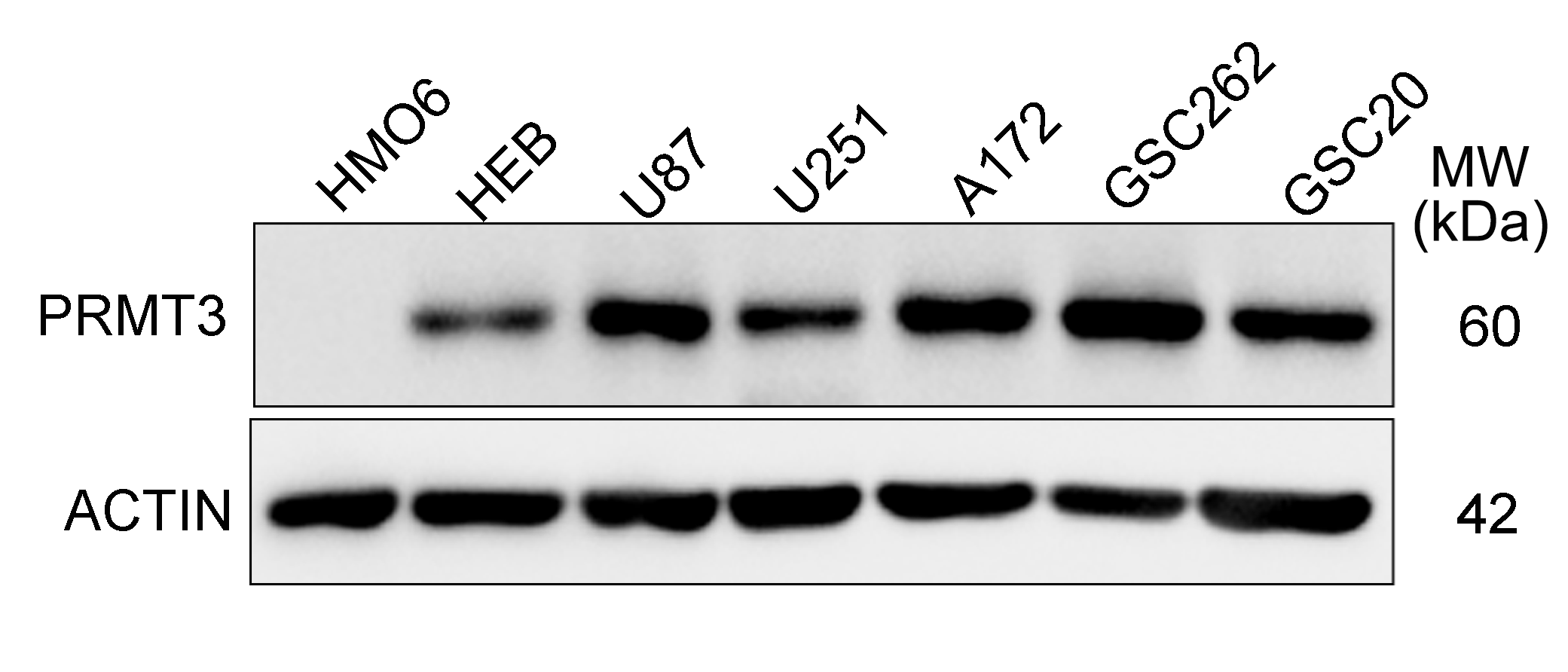

Supplement: Supplementary file 2 — Supplementary Figure1 [file 41419_2022_5389_MOESM2_ESM.tif]

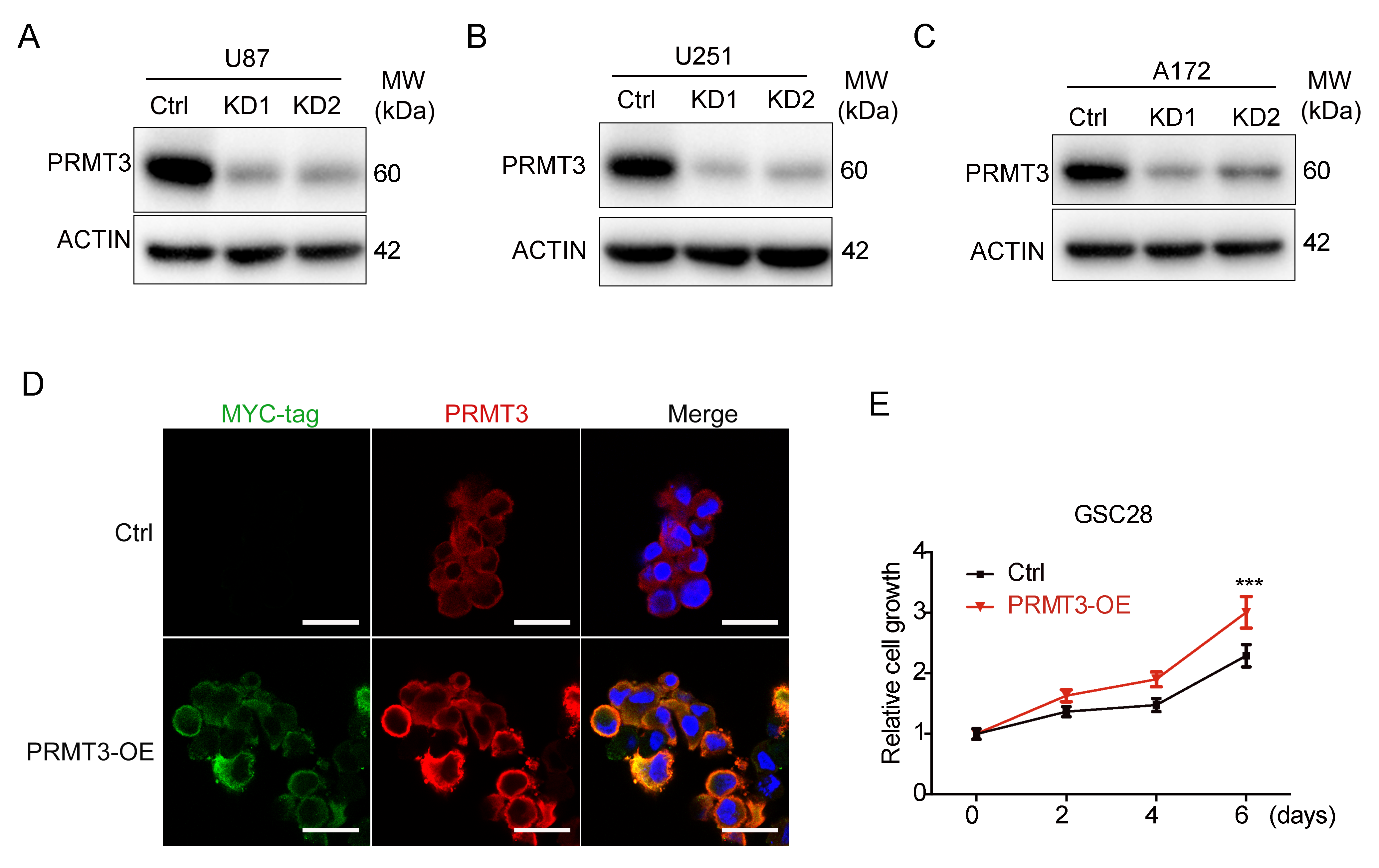

Supplement: Supplementary file 3 — Supplementary Figure2 [file 41419_2022_5389_MOESM3_ESM.tif]

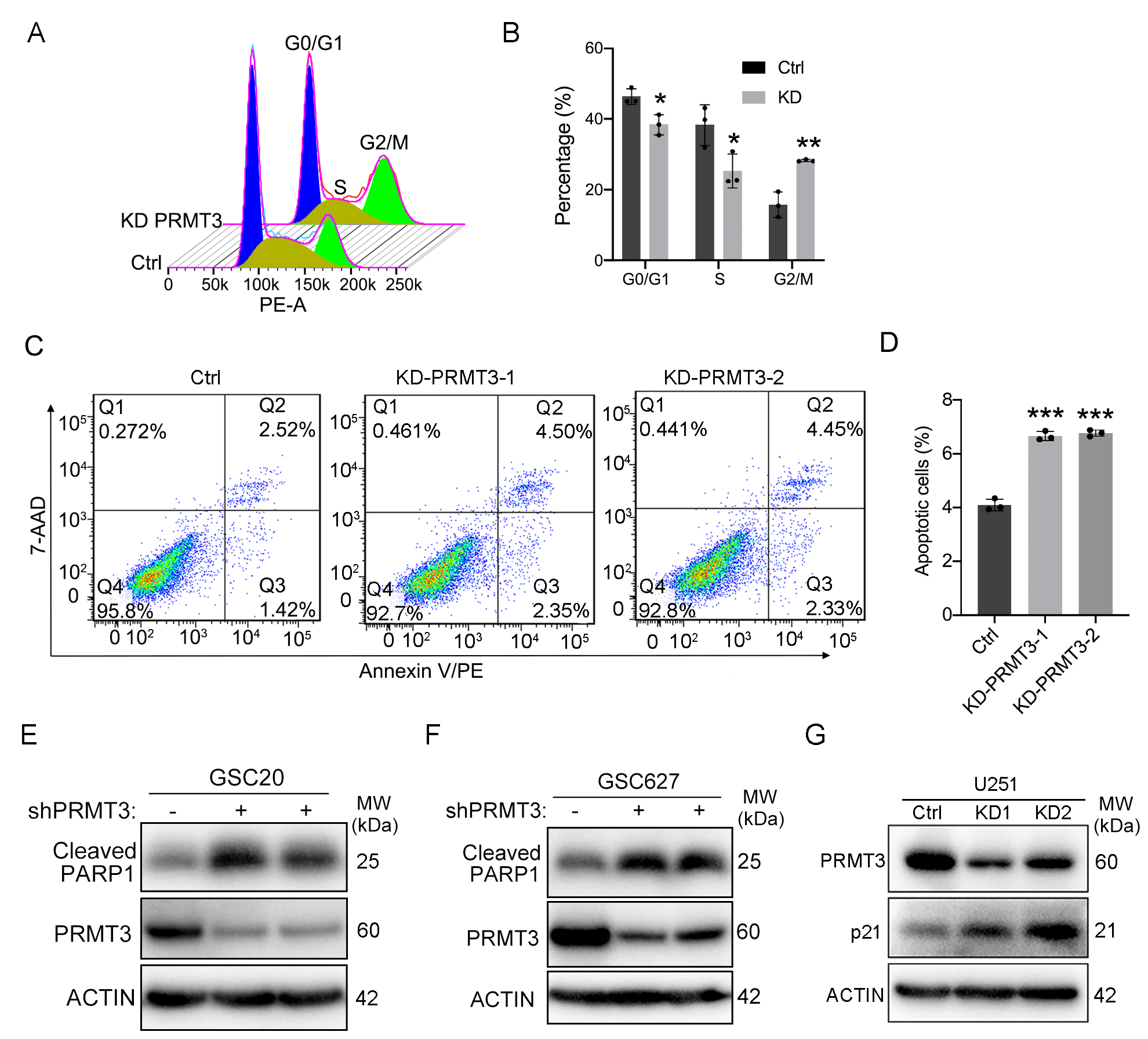

Supplement: Supplementary file 4 — Supplementary Figure3 [file 41419_2022_5389_MOESM4_ESM.tif]

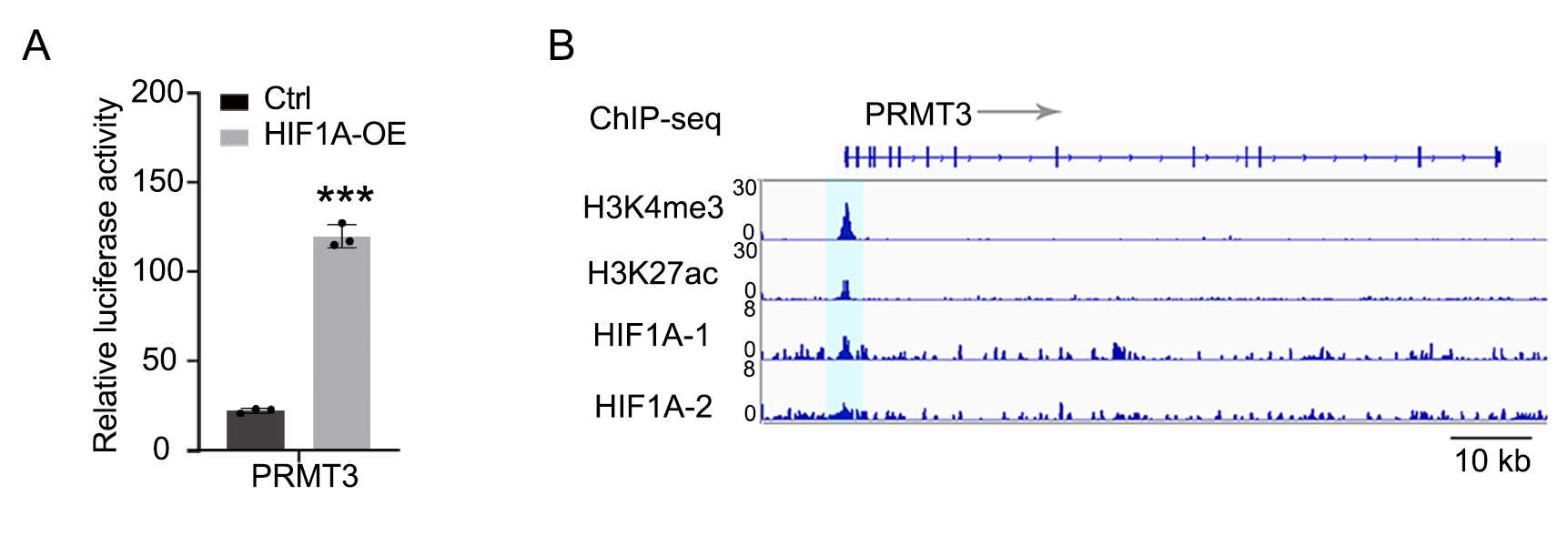

Supplement: Supplementary file 5 — Supplementary Figure4 [file 41419_2022_5389_MOESM5_ESM.tif]

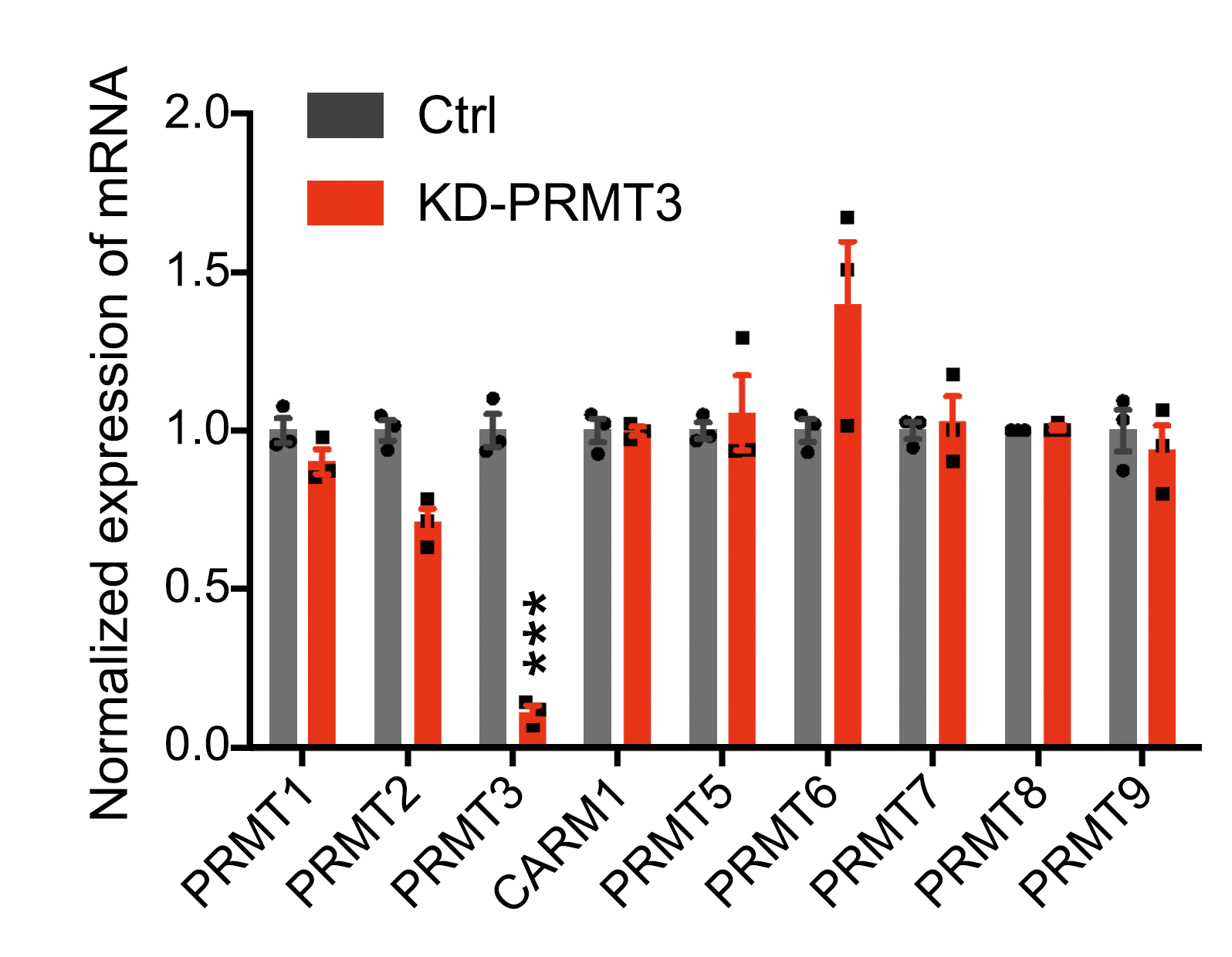

Supplement: Supplementary file 6 — Supplementary Figure5 [file 41419_2022_5389_MOESM6_ESM.tif]
