# Supplementary material for: PRMT3 drives glioblastoma progression by enhancing HIF1A and glycolytic metabolism
Source: Cell Death Dis. 2022 Nov 9;13(11):943. doi: 10.1038/s41419-022-05389-1 (PMC9646854; doi:10.1038/s41419-022-05389-1)

Figure 1F

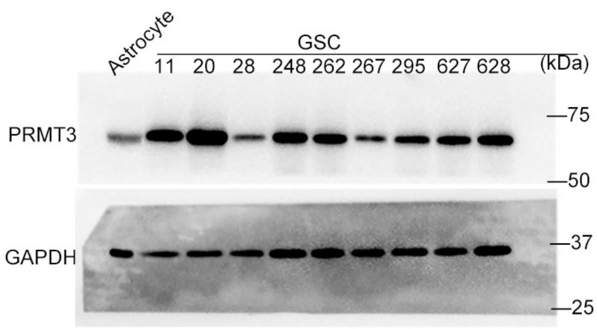

Supplementary Figure 1

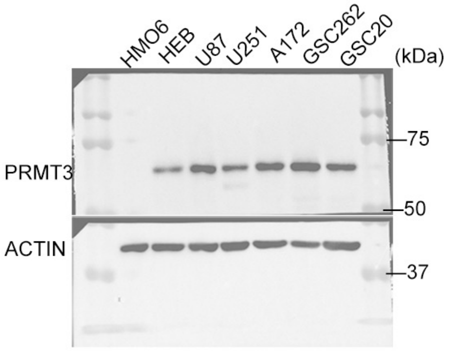

Figure 1H

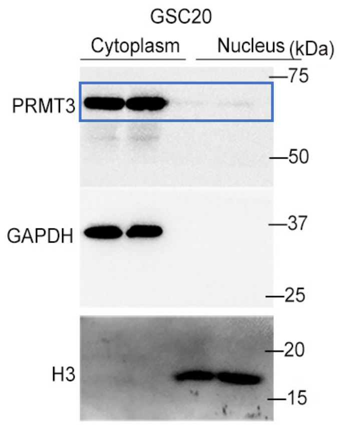

Supplementary Figure 2A

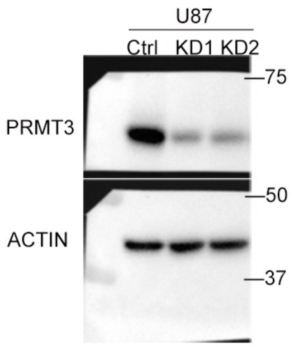

Supplementary Figure 2B

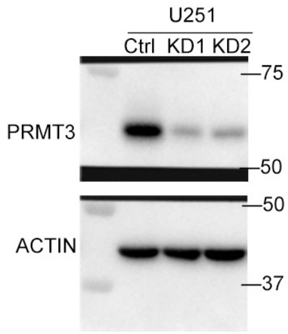

Supplementary Figure 2C

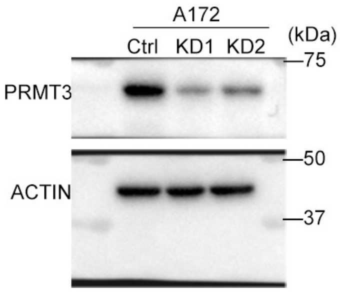

Figure 2F

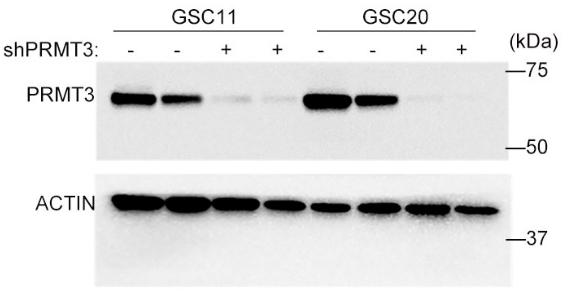

Figure 3E

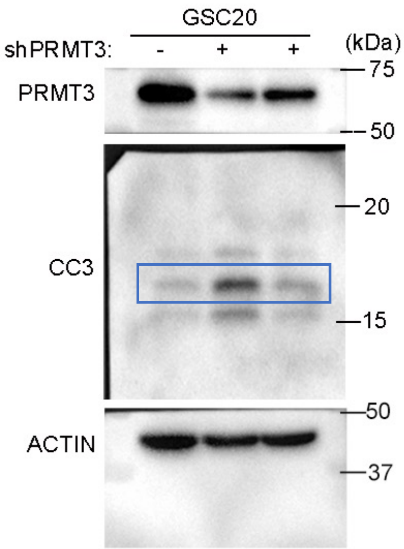

Figure 3F

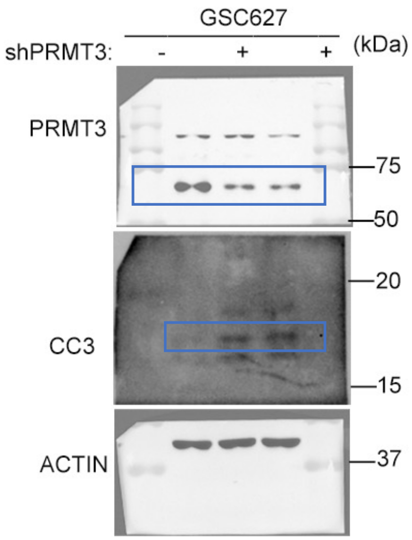

Figure 3G

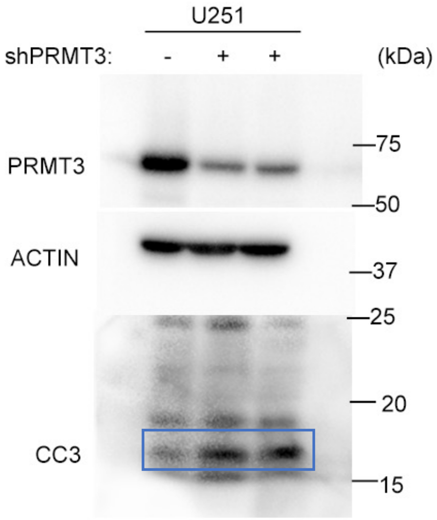

Supplementary Figure 3E

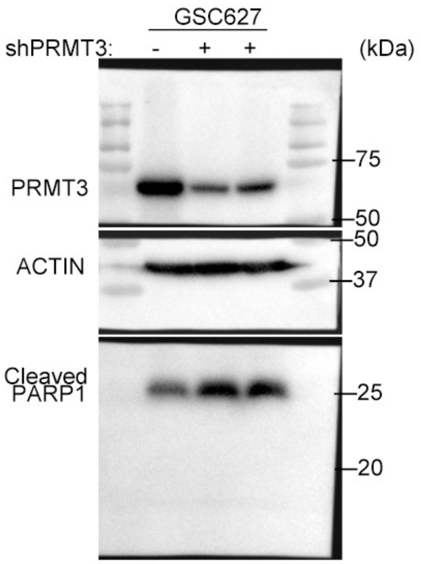

Supplementary Figure 3F

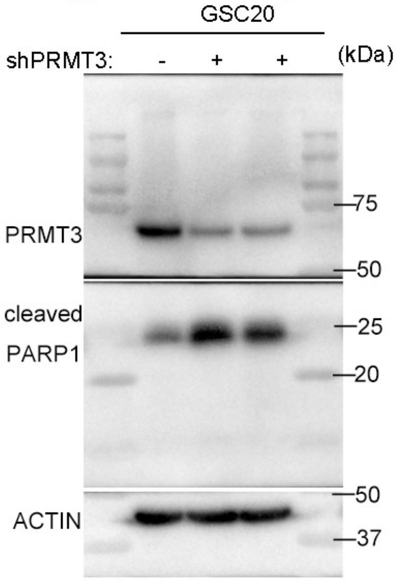

Supplementary Figure 3G

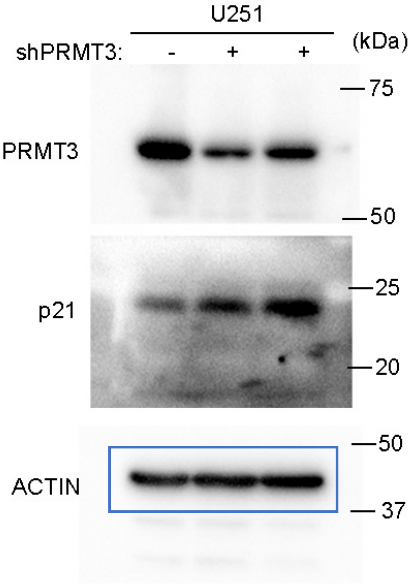

Figure 7C

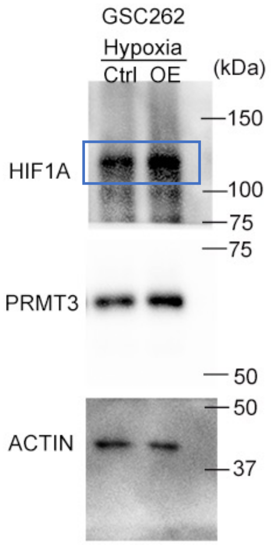

Figure 7D

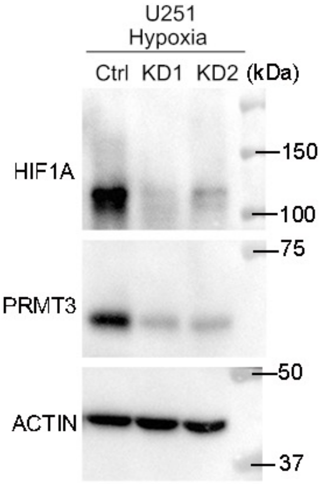

Figure 7E

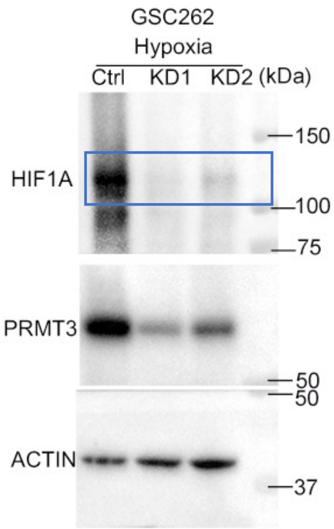

Figure7G

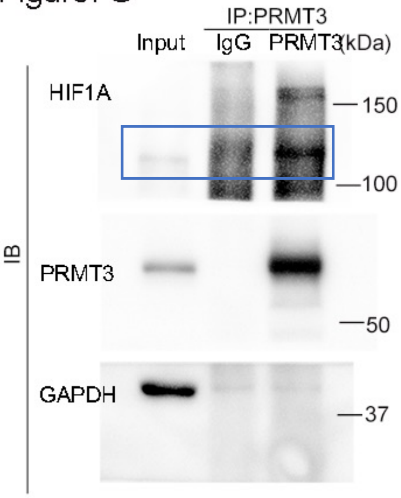

Figure 7F

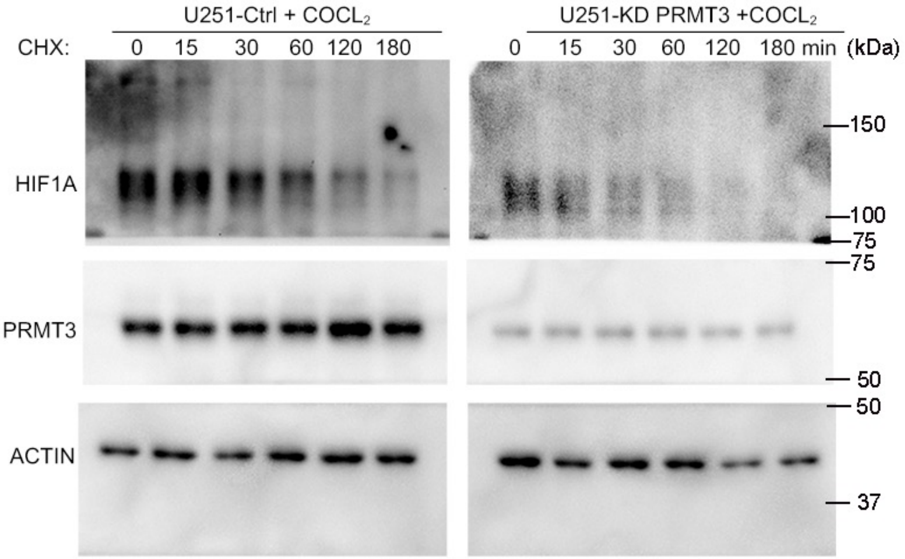

Figure 7H

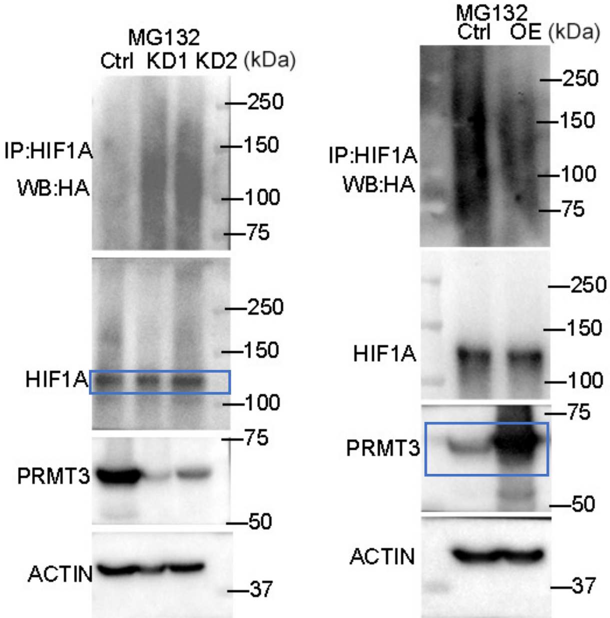

Supplement: Supplementary file 7 — Original Data File [file 41419_2022_5389_MOESM7_ESM.pdf]
